# Cytotoxic and Antimelanoma Activity of Selected 3-Methyl-1,6-diazaphenothiazines in Human Melanoma Cells—In Vitro Studies

**DOI:** 10.3390/cimb48050490

**Published:** 2026-05-09

**Authors:** Beata Morak-Młodawska, Małgorzata Jeleń, Zuzanna Rzepka, Milena Koch, Dorota Wrześniok

**Affiliations:** 1 Department of Organic Chemistry, Faculty of Pharmaceutical Sciences in Sosnowiec, Medical University of Silesia, Jagiellońska 4, 41-200 Sosnowiec, Poland; manowak@sum.edu.pl; 2 Department of Pharmaceutical Chemistry, Faculty of Pharmaceutical Sciences in Sosnowiec, Medical University of Silesia, Jagiellońska 4, 41-200 Sosnowiec, Poland; s83400@365.sum.edu.pl (M.K.); dwrzesniok@sum.edu.pl (D.W.)

**Keywords:** 1,6-diazaphenothiazines, melanoma cells, cytotoxicity, WST-1 assay, DNA damage, mitochondrial potential

## Abstract

The cytotoxic and mechanistic effects of novel 10-substituted 3-methyl-1,6-diazaphenothiazines were investigated in human melanoma models. Antiproliferative activity was evaluated in vitro using the WST-1 assay in four melanoma cell lines (A375, C32, G361, and SK-MEL-28) and normal human dermal fibroblasts (HDF). Among the tested derivatives, compound **6** exhibited the most pronounced biological activity, showing the strongest growth inhibition in melanoma cells, with the lowest IC_50_ value against C32 cells (54 µM), while displaying lower toxicity toward normal fibroblasts. Mechanistic studies using image cytometry and immunofluorescence revealed that compound **6** profoundly disrupts melanoma cell homeostasis by suppressing cell proliferation, inducing DNA damage, and activating apoptotic cell death. These effects were accompanied by mitochondrial membrane depolarization, depletion of intracellular reduced thiols, and DNA fragmentation, indicating the involvement of oxidative stress and mitochondrial dysfunction in the observed cytotoxic response. Taken together, these results demonstrate that 10-substituted 1,6-diazaphenothiazines exert anti-melanoma activity through multiple biological mechanisms. We believe our study provides a basis for developing derivatives with optimized pharmacological properties.

## 1. Introduction

Melanoma is a highly aggressive type of skin cancer associated with significant global mortality [1,2]. According to Arnold et al. [3], approximately 325,000 new cases and 57,000 deaths were recorded worldwide in 2020. If current trends continue, these numbers are expected to increase to about 510,000 cases and 96,000 deaths by 2040. The disease develops from melanocytes, the cells responsible for producing melanin, the natural pigment of the skin [4,5]. Melanoma can occur in two main forms: melanotic, characterized by a high melanin content, and amelanotic, in which the cells lose the ability to produce pigment [6,7]. Although several treatment approaches are currently used in melanoma therapy—including surgery, radiotherapy, chemotherapy, and immunotherapy—the search for new therapeutic strategies remains essential. This is largely due to the frequent development of both intrinsic and acquired drug resistance, which reduces the long-term effectiveness of existing treatments [8,9,10]. Therefore, there is a continuing need to identify novel compounds that can selectively target melanoma cells and potentially overcome resistance mechanisms.

As noted above, these challenges have prompted ongoing research efforts worldwide aimed at identifying new bioactive molecules with potential anticancer activity [11]. Among the wide range of organic compounds investigated, phenothiazines and their modified derivatives have attracted particular interest [12,13,14]. Depending on their chemical structure, phenothiazine-based compounds exhibit a broad spectrum of documented biological activities, including anticancer [15,16,17,18,19,20], antibacterial [21,22,23,24], antifungal [25,26], neuroprotective [13] and immunomodulatory effects [27,28]. This versatility makes phenothiazines a valuable scaffold in medicinal chemistry [29,30,31,32,33]. A distinct subgroup of modified phenothiazines is represented by dipyridothiazines, which are characterized by the presence of two pyridine rings incorporated into the phenothiazine core. Of the ten theoretically possible structural isomers of dipyridothiazines, only six (namely 1,6-, 1,8-, 1,9-, 2,7-, 3,6-, and 3,7-diazaphenothiazines) have been described in the literature to date [34]. Notably, many of these compounds have demonstrated significant cytotoxic activity against cancer cells, along with pronounced antioxidant and immunomodulatory properties. Mechanistic investigations into the anticancer activity of dipyridothiazine derivatives have revealed that their effects are primarily associated with activation of the mitochondrial apoptotic pathway. Gene expression analyses have confirmed their ability to disrupt the balance between anti-apoptotic BCL-2 and pro-apoptotic BAX proteins, thereby promoting apoptosis. In addition, some studies suggest that these compounds may exert their activity through DNA intercalation, further contributing to their cytotoxic effects [35,36]. Moreover, it has been shown that derivatives of this group have an affinity for deacetylase 6 and thus also activate the pathways of programmed cancer cell death [37].

**Figure 1:**
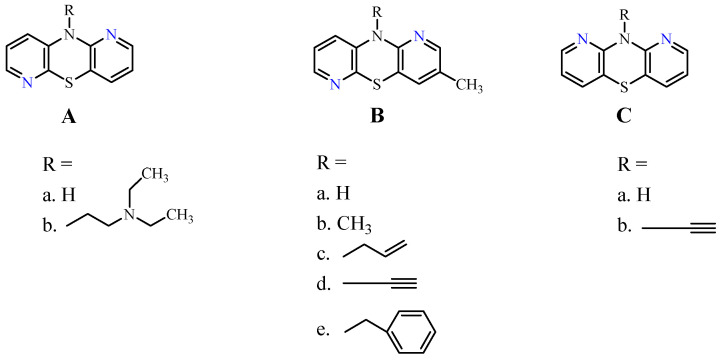
Diazaphenothiazine structures with promising cytotoxic activity against melanoma (figure created with ChemDraw—ChemOffice 23.1.2).

Among the many active diazaphenothiazines, selected derivatives presented in Figure 1 have shown promising cytotoxic activity against melanoma in previous studies.

The derivatives **Aa** and **Ab** depicted in Figure 1 displayed pronounced cytotoxic activity against C32 melanoma cells, with IC_50_ values in the range of 6.6–7.5 μg/mL [34,38]. Even greater potency was observed for derivatives **Ca** and **Cb**, which achieved IC_50_ values of approximately 3 μM, indicating a markedly enhanced anticancer efficacy [39]. In contrast, the **Ba**–**Be** derivative series displayed moderate cytotoxic activity, with IC_50_ values spanning 17–60 μM; however, these compounds demonstrated pronounced selectivity in comparative studies involving amelanotic and melanotic melanoma cell lines. This selectivity suggests that subtle structural modifications within the phenothiazine framework can significantly modulate both cytotoxic potency and cancer selectivity [40].

Structural analysis of the most active derivatives reveals a common structural motif, namely the presence of a nitrogen atom at position 1 of the tricyclic thiazine core. From a structure-activity relationship perspective, it is particularly noteworthy that the selected derivative **Ab** contains a dialkylaminoalkyl substituent at position 10. This functional group may act as a key pharmacophoric element, potentially enhancing cellular uptake, target interaction, or intracellular distribution, thereby contributing to the observed increases in anticancer potency and selectivity. Collectively, these findings identify phenothiazine-based derivatives as a promising class of compounds exhibiting selective cytotoxic activity against melanoma cells. The SAR trends provide a robust framework for rational molecular optimization and underscore the potential of this scaffold for further therapeutic development.

Based on the above observations, in the present article, we report the synthesis of a new series of dipyridothiazine derivatives, based on the **B**-core structure, functionalized with both selected labile dialkylaminoalkyl substituents and rigid heteroaromatic substituents containing electron-rich aromatic rings. These structural modifications introduce significant changes in the electronic and conformational properties of the molecules, substantially expanding the chemical space explored in our previous studies. The newly obtained compounds were evaluated for their cytotoxic activity against selected melanoma cell lines, and the results indicate a promising anticancer potential. Furthermore, preliminary studies on the underlying mechanisms of action were conducted, providing not only a biological characterization of the new derivatives but also initial insights into their molecular interactions with biological targets.

## 2. Materials and Methods

### 2.1. Chemistry

Commercial reagents and solvents used in the syntheses included: 3-dimethylaminopropyl chloride hydrochloride (96%), 2-diethylaminoethyl chloride hydrochloride (99.5%), 3-dimethylamino-2-methylpropyl chloride hydrochloride (99%), 4-chloro-3-nitropyridine, 2,6-dichloropyrazine, sodium hydroxide (Sigma-Aldrich, Burlington, MA, USA), DMF, dioxane, ethanol, and chloroform (POCh, Gliwice, Poland). 10*H*-3-methyl-1,6-diazaphenothiazine **1** was prepared according to a reported procedure [40].

^1^H NMR spectra were recorded on a Bruker AscendTM 600 spectrometer (600 MHz) in CDCl_3_ and DMSO_d6_ using tetramethylsilane (TMS) as the internal standard. ^13^C NMR spectra were recorded at 150 MHz on a Bruker AscendTM 600 spectrometer. The HRMS spectra (ESI—Electro Spray Ionisation) were run on a Brucker Impact II (Bruker, Billerica, MA, USA). The structure of the new compounds was documented in accordance with the recommended spectroscopic analyses [41]. Melting points of compounds **5** and **6** were measured in open capillary tubes on a Boetius melting point apparatus and are uncorrected.

### 2.2. Synthesis of 10-Dialkylaminoalkyl 3-Methyl-1,6-diazaphenothiazine Derivatives (***2***,***3***,***4***)

A mixture of 10*H*-3-methyl-1,6-diazaphenothiazine **1** (0.108 g, 0.50 mmol), sodium hydroxide (0.30 g, 7.5 mmol), and the appropriate dialkylaminoalkyl chloride hydrochloride (1.5 mmol; 2-diethylaminoethyl chloride hydrochloride, or 3-dimethylaminopropyl chloride hydrochloride, or 3-dimethylamino-2-methylpropyl chloride hydrochloride) in dry dioxane (10 mL) was heated at reflux for 5 h. After cooling to room temperature, the reaction mixture was poured into water (30 mL) and extracted with chloroform (3 × 30 mL). The combined organic extracts were dried over anhydrous Na_2_SO_4_ and concentrated under reduced pressure. The resulting yellow oils were purified by column chromatography on alumina using CHCl_3_ as the eluent to afford the following derivatives:

- 3-methyl-10-(2′-diethylaminoethyl)-1,6-diazaphenothiazine (**2**) (127 mg, 81%); yellow oil

^1^H NMR (CDCl_3_) δ (ppm): 1.20 (m, 6H, 2CH_3_), 2.17 (s, 3H, CH_3_), 2.81 (m, 4H, 2CH_2_), 2.92 (m, 2H, CH_2_), 4.20 (m, 2H, CH_2_), 6.76 (m, 1H), 7.02 (m, 1H), 7.19 (s, 1H, H), 7.94 (m, 2H). ^13^C NMR (CDCl_3_) δ (ppm): 10.9, 17.2, 29.7, 47.3, 116.5, 118.3, 120.8, 127.7, 134.3, 135.1, 144.1, 144.8, 149.9, 152.1. HRMS (EI) *m*/*z* for [C_17_H_22_N_4_S + H]^+^ calc. 315.1643, found: 315.1641.

b. 3-methyl-10-(3′-dimethylaminopropyl)-1,6-diazaphenothiazine (**3**) (114 mg, 76%); yellow oil

^1^H NMR (CDCl_3_) δ (ppm): 2.28 (m, 2H, CH_2_), 2.82 (s, 3H, CH_3_), 2.83 (s, 6H, 2CH_3_), 3.18 (m, 2H, NCH_2_), 4.12 (m, 2H, NCH_2_), 6.80 (m, 1H), 7.18 (m, 1H), 7.26 (s, 1H), 7.74 (m, 1H), 8.01 (m, 1H). ^13^C NMR (CDCl_3_) δ (ppm): 17.2, 21.9, 41.9, 42.1, 43.0, 55.9, 116.5, 118.6, 120.6, 128.1, 134.8, 138.1, 144.5, 144.8, 145.9, 152.1. HRMS (EI) *m*/*z* for [C_16_H_20_N_4_S + H]^+^ calc. 301.1487, found: 301.1503.

c. 3-methyl-10-(3′-dimethylamino-2′-methylpropyl)-1,6-diazaphenothiazine (**4**) (130 mg, 83%); yellow oil

^1^H NMR (CDCl_3_) δ (ppm): 1.05 (d, 3H, CH_3_), 2.19 (s, 3H, CH_3_), 2.34 (m, 8H, 2CH_3,_ CH_2_), 2.49 (m, 2H, CH_2_), 4.15 (m, 2H, *CH), 6.79 (m, 1H), 7.05 (m, 1H), 7.31 (m, 1H), 7.81 (s, 1H), 8.01 (m, 2H). ^13^C NMR (CDCl_3_) δ (ppm): 17.2,28.7, 45.6, 47.9, 64.3, 116.7, 118.5, 121.6, 129.3, 135.2, 138.1, 144.9, 145.1, 151.7. HRMS (EI) *m*/*z* for [C_17_H_22_N_4_S + H]^+^ calc. 315.1643, found: 315.1649.

### 2.3. Synthesis of 10-Heteroaryl 3-Methyl-1,6-diazaphenothiazine Derivatives (***5***,***6***)

To a solution of 3-methyl-10*H*-1,6-diazaphenothiazine **1** (0.15 g, 0.50 mmol) in dry DMF (5 mL) was added NaH (0.12 g, 5.0 mmol), and the reaction mixture was stirred at room temperature for 1 h. The appropriate chloroheteroaryl derivative (2,6-dichloropyrazine or 4-chloro-3-nitropyridine) (1.5 mmol) was then added, and stirring was continued for 24 h. The reaction mixture was poured into water (25 mL), and the resulting precipitate was collected by filtration, washed with water, and purified by column chromatography on alumina using CHCl_3_ as the eluent to afford the following derivatives:

- 3-methyl-10-(6-chloropyrazin-2-yl)-1,6-diazaphenothiazine (**5**) (115 mg, 71%); m.p. 213–214 °C.

^1^H NMR (CDCl_3_) δ (ppm): 2.36 (s, 3H, CH_3_), 7.31 (m, 1H), 7.56 (m, 1H), 8.05 (m, 1H), 8.11 (m, 1H), 8.29 (s, 1H), 8.32 (m, 1H), 8.89 (s, 1H). ^13^C NMR (CDCl_3_) δ (ppm): 17.8, 112.8, 122.7, 126.7, 127.9, 132.3, 133.2, 135.8, 137.7, 137.9, 144.2, 145.9, 148.8, 149.5, 154.8. HRMS (EI) *m*/*z* for [C_15_H_10_ClN_5_S + H]^+^ calc. 328.0424, found: 328.0419.

b. 3-methyl-10-(3-nitropyridin-4-yl)-1,6-diazaphenothiazine (**6**) (115 mg, 68%); m.p. 207–209 °C.

^1^H NMR (DMSO_d6_) δ (ppm): 2.5 (s, 3H, CH_3_), 6.95 (s, 2H), 7.13 (s, 1H), 7.61 (s, 1H), 7.76 (m, 1H), 7.94 (d*,* J *=* 5.1Hz, 1H), 8.82 (d, J *=* 5.1Hz, 1H), 9.25 (s, 1H). ^13^C NMR (CDCl_3_) δ (ppm): 17.2, 112.8, 120.8, 123.1, 126.9, 127.3, 135.1, 136.3, 137.7, 139.9, 142.4, 144.6, 145.6, 147.0, 149.5, 154.5. HRMS (EI) *m*/*z* for [C_16_H_11_N_5_O_2_S + H]^+^ calc. 338.0712, found: 338.0705.

### 2.4. Evaluation of Anticancer Activity in Melanoma Cells

#### 2.4.1. Cell Culture

A375, G361, SK MEL-28 and C32 melanoma cell lines were obtained from ATCC (ATCC, Manassas, VA, USA). Human Dermal Fibroblasts were purchased from Sigma-Aldrich (St. Louis, MO, USA). Cells were incubated at 37 °C in a humidified atmosphere containing 5% CO_2_. Cultures were maintained in the appropriate medium (DMEM for A375, C32, SK-MEL-28; McCoy’s 5A for G361) supplemented with 10% heat-inactivated fetal bovine serum. To prevent microbial contamination, penicillin-streptomycin (10,000 U/mL) solution was added to the culture medium (1:100). Treatments with the analyzed chemical compounds were initiated 24 h after cell seeding.

#### 2.4.2. Cytotoxicity Screening

Cell viability was assessed using the WST-1 assay. In this assay, the water-soluble tetrazolium salt is reduced to formazan, a water-soluble dye with a maximum absorption at 440 nm. Cells were seeded into 96-well plates at a density of 3000 cells per well and incubated for 24 h. The culture medium was then replaced with solutions of each compound at the following concentrations: 10, 25, 50, 75, and 100 µM. Cells were incubated for an additional 72 h. Two hours before the end of the incubation, 10 µL of WST-1 reagent was added to each well. Absorbance was measured using a microplate reader (Infinite 200 Pro, TECAN, Männedorf, Switzerland) at 440 nm and 660 nm.

#### 2.4.3. Cell Count Assay

Cell counts were determined using the NucleoCounter NC-3000 cytometer (ChemoMetec, Lillerød, Denmark) with the NucleoView software (version 1.4), following the manufacturer’s protocol. Briefly, detachment of cells was performed with the addition of trypsin/EDTA solution to cell cultures in a T-75 flask. After 24 h, cells were centrifuged and resuspended in dedicated medium. Samples of cell suspensions were loaded into Via1-Cassettes containing DAPI and acridine orange, dyes that differentially stain cells according to their viability.

#### 2.4.4. Assessment of the Level of Cellular Reduced Thiols

The level of cellular reduced thiols was measured using the NucleoCounter NC-3000 following the manufacturer’s protocol. Cell suspensions were stained with solution 5, containing acridine orange, propidium iodide, and VitaBright-48 (VB48), which stains cells with an intensity proportional to the level of reduced thiols. Suspensions were loaded into NC-Slides A8 and analyzed. Histograms used to visualize the results were obtained using NucleoView NC-3000 software.

#### 2.4.5. Mitochondrial Potential Assay

Mitochondrial membrane potential was assessed using the JC-1 dye. In cells with high membrane potential, JC-1 cations form aggregates in mitochondria, emitting red fluorescence, whereas in cells with low potential, the dye remains in the cytoplasm and emits green fluorescence. After treatment with the tested compounds, cells were incubated with solution 7 containing JC-1 for 15 min at 37 °C and then washed with PBS. Cell pellets were resuspended in solution 8 containing DAPI. Suspensions were loaded into NC-Slides A8 and analyzed. Scatter plots were generated using the NucleoView NC-3000 software.

#### 2.4.6. DNA Fragmentation Assay

The NucleoCunter NC-3000 image cytometer was used to assess fragmentation of DNA. In brief, cells were fixed with ice-cold ethanol, washed with PBS, and then stained with solution 3, a mixture of DAPI and Triton X-100. Suspensions were loaded into 8-chamber NC-Slides and analyzed using the cytometer and NucleoView software. Histograms were obtained using the NucleoView NC-3000 software.

#### 2.4.7. Phospho-Histone H2AX Immunofluorescence Assay

Phospho-Histone H2AX (Ser139) immunofluorescence assay was used to evaluate DNA double-strand breaks induced by the tested compound in melanoma cells. Imaging of cells was performed using the laser confocal microscope Nikon Eclipse Ti-E A1R-Si and Nikon NIS Elements AR software (Ver 4.51.00, Nikon Instruments, Tokyo, Japan). Cells were seeded and cultured on coverslips. After the treatment, the cells were fixed with 4% paraformaldehyde. In the next step, the samples were permeabilized with 0.1% Triton X-100, incubated with 0.25% glycine and 3% bovine serum albumin solutions. Then the samples were incubated with γ-histone H2AX (Ser139) rabbit antibody (1:500) overnight. Subsequently, the cells were stained with Alexa Fluor 488 conjugated with the secondary anti-rabbit antibody (1:200) and Phalloidin-Atto 565 (300 nM) to visualize the target protein and actin filaments, respectively.

#### 2.4.8. Statistical Analysis

Statistical analysis was performed using GraphPad Prism 8.0.1 software. All experiments were performed at least in triplicate. Data are presented as mean ± standard deviation (SD). Statistical analysis was conducted using one-way analysis of variance (ANOVA) followed by Dunnett’s post hoc test to compare treated groups with the control. Differences were considered statistically significant at *p* < 0.05.

## 3. Results

### 3.1. Chemical Part

**Scheme 1:**
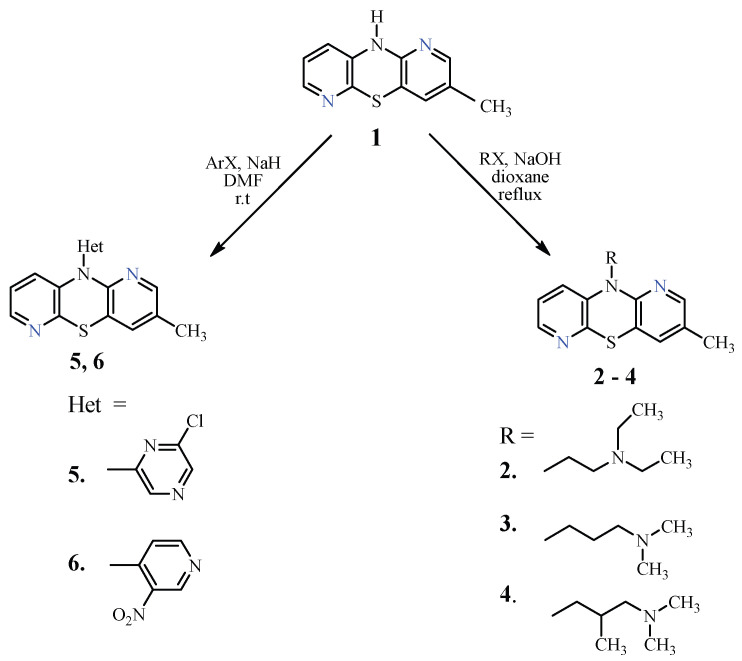
Synthetic pathways for new 10-substituted 3-methyl-1,6-diazaphenothiazines **2**–**6** (scheme created with ChemDraw—ChemOffice 23.1.2).

The synthesis of the starting material, 3-methyl-10*H*-1,6-diazaphenothiazine **1**, has been reported previously, and its structure was confirmed using 2D NMR spectroscopy and mass spectrometry [40]. A series of 10-substituted 3-methyl-1,6-diazaphenothiazines bearing dialkylaminoalkyl (**2**–**4**) or heteroaryl (**5**,**6**) groups at the thiazine nitrogen were synthesized (Scheme 1). Compounds **2**–**4** were obtained via base-promoted N-alkylation using alkylaminoalkyl chloride hydrochlorides in refluxing dioxane with sodium hydroxide, while heteroaryl derivatives were prepared by N-arylation with sodium hydride in anhydrous DMF. All reactions proceeded with good yields, and the structures were confirmed by NMR and HRMS (Section 2.1). The spectra of compound **6**, which exhibited the highest biological activity, are provided in the Supplementary Materials.

### 3.2. Effects of Novel Derivatives on Melanoma Cells

#### 3.2.1. Cytotoxicity Screening

**Figure 2:**
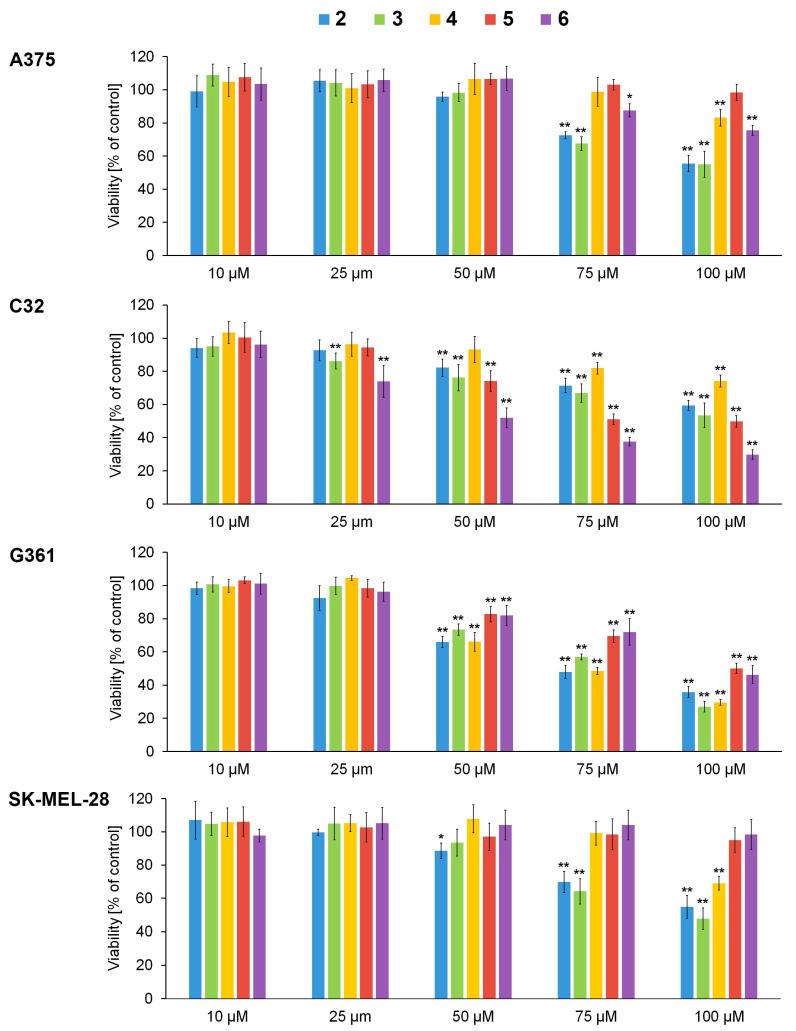
Effects of derivatives **2**–**6** on the viability of melanoma cells. The A375, C32, G361, and SK-MEL-28 cell lines were incubated with the compounds at concentrations of 10–100 µM for 72 h prior to the WST-1 assay. Data are presented as mean ± SD from three independent experiments; * *p* < 0.05 and ** *p* < 0.01 relative to the control (the control value was set to 100%).

**Figure 3:**
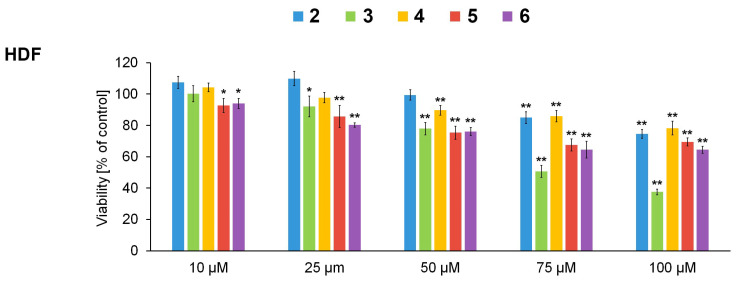
Effects of derivatives **2**–**6** on the viability of human dermal fibroblasts (HDF). The cells were incubated with the compounds at concentrations of 10–100 µM for 72 h prior to the WST-1 assay. Data are presented as mean ± SD from three independent experiments; * *p* < 0.05 and ** *p* < 0.01 relative to the control (the control value was set to 100%).

The preliminary WST-1 assay results for derivatives **2**–**6** in four melanoma cell lines (A375, C32, G361, and SK-MEL-28) are shown in Figure 2, representing the first step of the in vitro evaluation. Cells were exposed to increasing concentrations of the compounds (10–100 µM) for 72 h. To evaluate selective toxicity, parallel experiments were performed on normal human dermal fibroblasts (HDF), allowing assessment of potential effects on healthy cells (Figure 3). IC_50_ values, representing the concentrations required to reduce cell viability by 50% compared to untreated controls, were calculated based on the observed results (Table 1). Based on the screening results (Figure 2 and Figure 3), compound **6** was chosen for further studies due to its strong activity against C32 cells and lower toxicity toward HDF cells.

#### 3.2.2. Cell Count Assay

**Figure 4:**
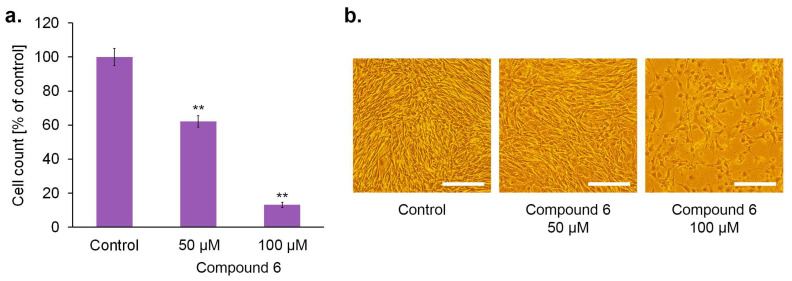
Impact of the derivative **6** on the population dynamics of C32 melanoma cells. (**a**) The results from the cell count assay after the treatment with the compound at concentrations of 50 or 100 µM for 72 h. Data are presented as mean ± SD from three independent experiments; ** *p* < 0.01 relative to the control. (**b**) Representative micrographs illustrating the C32 cells after the treatment. Images were acquired using a phase-contrast microscope at 10× magnification, scale bar = 200 µm.

Cytometric analysis was performed to determine the impact of compound **6** on the number of C32 cells following 72 h of incubation. Concentrations of 50 µM (≈IC_50_) and 100 µM (2 × IC_50_) were used. The findings, presented in Figure 4a, were supported by phase-contrast microscopic observations (Nikon Eclipse TS100-F) highlighting differences between treated and control cells (Figure 4b).

#### 3.2.3. Evaluation of Intracellular Reduced Thiols and Mitochondrial Potential

**Figure 5:**
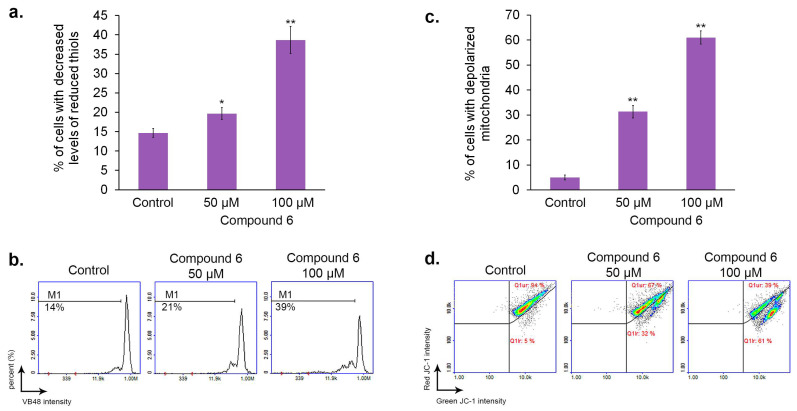
Impact of derivative **6** on intracellular reduced thiols levels (**a**,**b**) and mitochondrial potential (**c**,**d**) in C32 melanoma cells. Cells were incubated with the compound at concentrations of 50 or 100 µM for 72 h prior to cytometric analysis. (**a**,**c**) Bar graphs presenting data as mean ± SD from three independent experiments, * *p* < 0.05, ** *p* < 0.01 relative to the control. (**b**) Representative histograms, where M1 corresponds to the percentage of cells with low levels of reduced thiols. The arrows on the x-axis in (**b**) indicate the gating thresholds used by the NucleoView software to distinguish between different levels of DNA fragmentation. (**d**) Representative scatter plots, where Q1lr corresponds to the percentage of cells with depolarized mitochondria, and Q1ur corresponds to the percentage of cells with polarized mitochondria. On the scatterplot, the colors represent the value intensity from low to high. Blue indicates the lowest values, while red represents the highest values.

The redox balance of C32 melanoma cells treated with compound **6** was assessed by determining their intracellular content of reduced thiols after 72 h of incubation with the compound at concentrations of 50 or 100 µM. The results are presented in Figure 5a. Additionally, the mitochondrial potential of the tested cells was evaluated using image cytometry, and the results are demonstrated in Figure 5b.

#### 3.2.4. Assessment of DNA Fragmentation and γ-H2AX Levels

**Figure 6:**
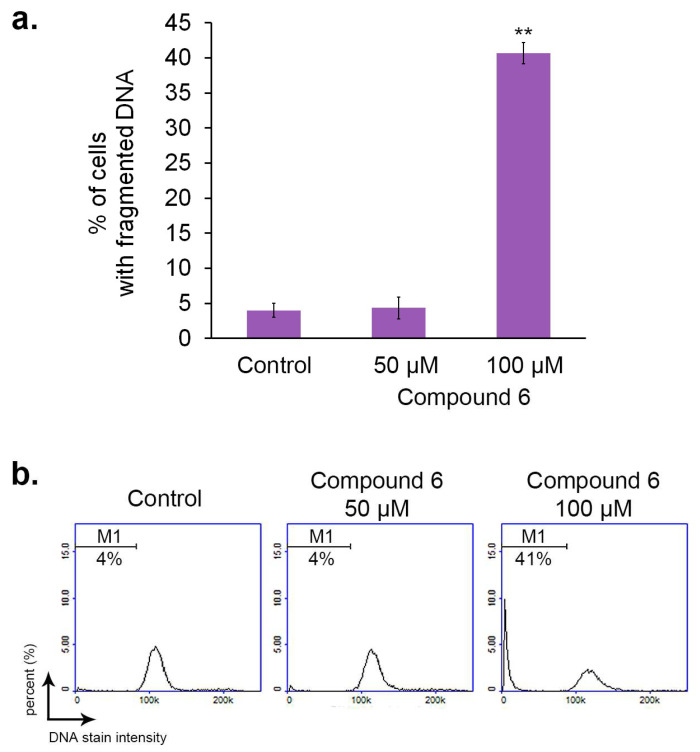
Analysis of DNA fragmentation in C32 cells treated with compound **6**. Cells were incubated with the compound at concentrations of 50 or 100 µM for 72 h prior to cytometric analysis. (**a**) Bar graphs presenting data as mean ± SD from three independent experiments, ** *p* < 0.01 relative to the control. (**b**) Representative histograms, where M1 corresponds to the percentage of cells with fragmented DNA.

**Figure 7:**
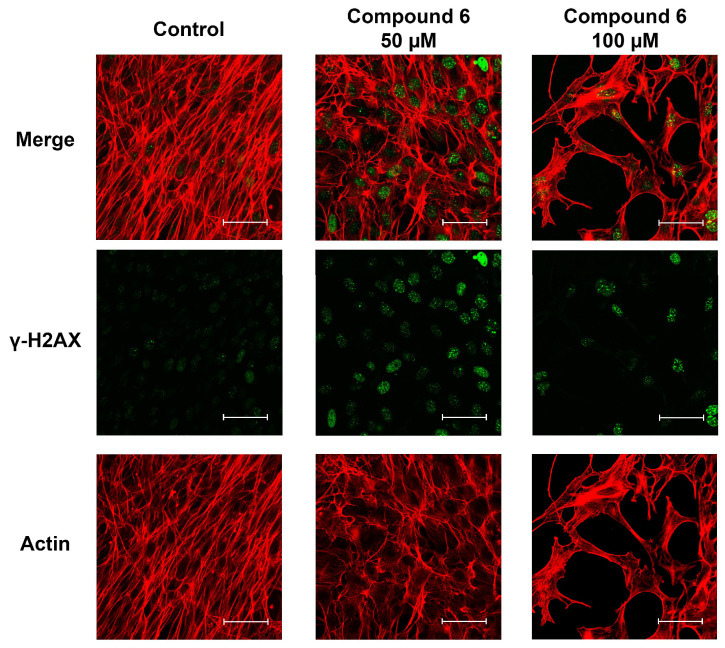
Comparison of γ-H2AX levels in control C32 melanoma cells and cells treated with the **6** derivative for 72 h. The figure presents merged confocal images (green—immunolabeled γ-H2AX; red—actin filaments) as well as the separate channels. Scale bar 50 µm.

The next step involved evaluating DNA fragmentation in the C32 melanoma cell population treated with compound **6** at concentrations of 50 and 100 μM, with the results shown in Figure 6. Additionally, the level of the double-strand DNA damage marker γ-H2AX was assessed by immunofluorescence, and the findings of this analysis are presented in Figure 7.

## 4. Discussion

### 4.1. Chemical Part

Phenothiazines are typically synthesized via pathways involving the Smiles rearrangement, with the reaction outcome strongly influenced by the substrate structure and the reaction conditions. In certain cases, monitoring the rearrangement can be challenging, as the rearranged and non-rearranged products may be identical or differ only subtly, for instance, in substituent positions or the nitrogen atom location, as observed for aza-phenothiazines [34]. The synthesis of the starting material, 3-methyl-10*H*-1,6-diazaphenothiazine **1**, has been reported previously, and its structure was confirmed using 2D NMR spectroscopy and mass spectrometry [40]. In the present study, we synthesized a series of 10-substituted 3-methyl-1,6-diazaphenothiazines to increase the structural diversity by including labile dialkylaminoalkyl groups (**2**–**4**) and strained heteroaryl rings **5**,**6** functionalized with chloride and nitro substituents on the thiazine nitrogen atom (Scheme 1). Derivatives **2**–**4** were obtained via base-promoted N-alkylation of the thiazine nitrogen using the corresponding alkylaminoalkyl chloride hydrochlorides in refluxing dioxane with sodium hydroxide. In contrast, heteroaryl derivatives were achieved through N-arylation under strongly basic conditions using sodium hydride in anhydrous DMF and the appropriate chloroheteroaryl. All reactions proceeded efficiently, yielding the target compounds in good yield. The product structures were confirmed by NMR spectroscopy and high-resolution mass spectrometry HRMS.

### 4.2. Biological Part

Building on our previous work [40] demonstrating the anticancer activity of 3-methyl-1,6-diazaphenothiazines, the newly synthesized derivatives were evaluated for their preliminary effects on melanoma cell viability. The investigation aimed to assess how structural modifications at the thiazine nitrogen, including dialkylaminoalkyl and heteroaryl substituents, influence cytotoxic activity, providing insight into structure–activity relationships relevant to their mechanism of action.

The cytotoxic effects of derivatives **2**–**6** on four human melanoma cell lines (A375, C32, G361, and SK-MEL-28) were evaluated using the WST-1 assay. Cells were treated with increasing concentrations of the compounds (10–100 µM) for 72 h. The cytotoxicity study also included experiments on normal human dermal fibroblasts (HDF) to assess whether the compounds are selectively toxic to cancer cells without affecting healthy tissue (Figure 2 and Figure 3). Based on the obtained results, the IC_50_ values were determined, representing the concentrations that caused a 50% reduction in cell viability relative to the untreated control (Table 1). As shown in Figure 2, compounds **2** and **3** significantly reduced the survival of all melanoma cell lines tested at concentrations of 75 and 100 μM. G361 cells were the most sensitive to the cytotoxicity of compounds **2** and **3**. However, it should be noted that compound **3** also significantly reduced the survival of normal cells (Figure 3), while compound **2** was more selective towards cancer cells. Compound **4** had a strong effect only on G361, but was less toxic to normal cells compared to **2** and **3**. Derivatives **5** and **6** exhibited cytotoxic effects on melanoma cell lines C32 and G361, without showing high toxicity towards HDF. The effect of derivative **6** on C32 melanoma cells deserves special attention, as at concentrations ranging from 25 to 100 µM, it caused a concentration-dependent reduction in cell survival, with an IC_50_ of approximately 54 µM (Table 1). The IC_50_ value of compound **6** indicates moderate cytotoxicity compared to highly potent cytostatic agents. For example, cisplatin exhibits strong activity against C32 melanoma cells at concentrations as low as 0.3 µM [42], while oxaliplatin shows an IC_50_ of 0.98 µM after 24 h incubation [43]. In contrast, alkylating agents such as temozolomide (TMZ) and dacarbazine (DTIC) display relatively weak activity in melanoma models (IC_50_ values of TMZ and DTIC in A375 cells after 72 h were found to be 943 µM and 1113 μM, respectively [44]).

When evaluating new anticancer candidates, selectivity toward cancer cells is a critical parameter influencing potential in vivo safety. Compound **6** showed a selectivity index (SI = IC_50_ normal cells/IC_50_ cancer cells) of 3.48, indicating a moderate preference for C32 melanoma cells over normal cells. Although this indicates some degree of differential activity, the observed selectivity remains limited and warrants further investigation. However, this result suggests that compound **6** may serve as a promising scaffold for further optimization toward improved potency and selectivity.

Present results are consistent with several previous works that indicate that structural variants of phenothiazine and related compounds can exert cytotoxic effects in a variety of cancer cell lines [33,36,37,38,39,45]. Our previous studies on the effect of 3-methyl-1,6-diazaphenothiazines on melanoma cells have shown that this group of compounds includes derivatives with a promising activity profile that are more cytotoxic to melanoma cells than to normal cells [40]. In the aforementioned study, for the C32 line, we demonstrated that the derivative 3-metyhl-10-allyl-1,6-diazaphenothiazine Bc and 3-methyl-10-benzyl-1,6-diazaphenothiazine Be had an IC_50_ of 59.6 µM and 42.68 µM, respectively, thus exhibiting similar activity to the derivative **6** presented in this study. Apart from compound **3**, the series of derivatives tested in this study exhibits significantly lower cytotoxicity towards normal fibroblasts (Figure 2 and Table 1) compared to the 3-methyl-1,6-diazophenothiazine derivatives Ba–Be evaluated in our previous studies [40]. This information is critical for guiding future structural optimization of compounds within this chemical class. Considering the results of the screening analysis (Figure 2 and Figure 3 and Table 1), compound **6** was selected for further analysis because it exhibited the most promising selectivity index compared to the other compounds. A concentration of the compound close to the IC_50_ value (50 µM) was used, as well as a concentration equal to twice that value (100 µM).

The first stage of the cytometric analysis involved assessing the influence of compound **6** on the size of the C32 cell population. We demonstrated that the tested compound inhibits melanoma cell proliferation in a concentration-dependent manner—at a concentration of 50 µM, the total number of cells was approximately 40% lower compared to the untreated cells (control), while after incubating cells with 6 at a concentration of 100 µM, the number of cells was only 13% of the control value (Figure 4a). Observations under a phase contrast microscope (Nikon Eclipse TS100-F) confirmed the differences in cell count between the control culture and cultures treated with compound **6**, as determined by cytometry (Figure 4b).

Thiol-containing molecules, particularly glutathione (GSH), constitute a major defense system against oxidative cytotoxicity, and lowering GSH levels promotes apoptosis and necrosis [46,47]. In this study, the intracellular level of reduced thiols was assessed to determine the redox homeostasis of melanoma cells treated with compound **6**. As shown in Figure 5a,b, incubation of cells with **6** derivatives increases the percentage of cells with depleted reduced thiols pool. In the case of C32 cells exposed to the compound at a concentration of 100 µM, cells with low thiol levels accounted for approximately 40% of the population, whereas in the control population, this percentage was approximately 14%. The observed changes indicate alterations in the antioxidant capacity of C32 melanoma cells, suggesting that the cytotoxic effect of compound **6** may be associated with oxidative stress–mediated mechanisms, including glutathione depletion. In our previous study [40], we observed a similar effect of 3-methyl-1,6-diazaphenothiazine derivatives **Bc** and **Be** in the C32 cell line, suggesting that the tested derivatives may share a comparable mechanism of cytotoxicity.

Since mitochondria are highly sensitive to oxidative stress, GSH depletion can compromise mitochondrial function, leading to loss of membrane potential. To investigate whether the observed decrease in reduced thiols affects mitochondrial integrity, we measured the mitochondrial membrane potential of C32 cells after treatment with **6** derivative. The results are presented in Figure 5c. In our experiments, we observed a significant decrease in mitochondrial potential (Δψm) in C32 cells after exposure to the compound. The percentage of cells with depolarised mitochondria was approximately 30% in melanoma cells treated with **6** at a concentration of 50 μM or 60% in cells treated with a 100 μM solution of **6**. Mitochondrial depolarisation indicates a disruption of the internal integrity of the mitochondrial membrane, which may lead to the loss of the proton gradient necessary for ATP generation and proper functioning of the respiratory chain. This may result in the activation of mitophagy or cell death, including apoptosis [48,49]. In our previous study on 3-methyl-1,6-diazaphenothiazine derivatives [40], we demonstrated that compounds with allyl and benzyl substituents also caused mitochondrial depolarisation in C32 cells, but this effect was significantly weaker than that observed in the present study for derivative **6** containing a pyridine ring with an electron-withdrawing nitro substituent. A similar phenomenon was described in a study on the C32 line after treatment with minocycline and doxycycline. In this study, at the highest concentrations, the percentage of C32 cells with low Δψm increased to approximately 40–50%. In addition, a simultaneous increase in caspase activation and cytochrome c release was observed, indicating the involvement of the intrinsic apoptosis pathway [50]. Therefore, the mitochondrial depolarisation observed in the present study may suggest that **6** derivative likely acts as a stressor for mitochondria, impairing their function and leading to the activation of cell death mechanisms. Nevertheless, this hypothesis requires further in-depth research.

DNA fragmentation is a process involving the controlled or uncontrolled breakdown of genetic material into smaller fragments. It is a key stage of apoptosis, where chromatin degradation occurs in an orderly manner under the influence of endonucleases. In the case of genotoxic stress or necrosis, fragmentation can be uncontrolled and result from massive DNA damage [51,52]. In this study, we observed a significant percentage (approximately 40%) of cells with DNA fragmentation in the C32 melanoma cell population treated with compound **6** at a concentration of 100 μM (Figure 6). For derivative **6** at a concentration of 50 µM, the percentage of cells with DNA fragmentation did not increase compared to the control group. In our earlier work on anti-melanoma potential of diazaphenothiazines [40] we showed that dipyridothiazinee with benzyl substituent Be also caused DNA fragmentation in C32 melanoma cells, but the effect was significantly weaker than for derivative **6**.

The last stage of our in vitro experimental panel was immunofluorescence staining of γ-H2AX (histone H2AX phosphorylated at Serine 139), which is used to detect DNA damage, especially double-strand breaks (DSB) [53]. As shown in Figure 7, microscopic observations revealed that the γ-H2AX level was higher in C32 cells incubated with 6 compared to the control sample. Therefore, the increased γ-H2AX levels and DNA fragmentation (Figure 6) demonstrated by cytometry strongly suggest that DNA damage in tested cells exceeded the repair threshold, leading to the activation of mechanisms resulting in cell death—apoptosis or another form of DSB-dependent cell death.

It should also be noted that the cytotoxic effects of compound **6** were observed at relatively high concentrations (IC_50_ ≈ 50 µM). It is important to emphasize that the present study represents a preliminary stage in the exploration of 10-substituted 3-methyl-1,6-diazaphenothiazines. The aim was not to identify a definitive anticancer drug candidate, but rather to provide an initial assessment of biological activity and to establish a basis for further structural optimization.

## 5. Conclusions

The synthesis of novel 10-substituted 3-methyl-1,6-diazaphenothiazines obtained *via* alkylation and heteroarylation is presented. The structures of these derivatives were confirmed by NMR spectroscopy and high-resolution mass spectrometry (HRMS). The compounds were subsequently evaluated for cytotoxic activity against melanoma cells and normal human fibroblasts. Cytotoxicity screening in four melanoma cell lines revealed that compounds **2**, **3**, and **4** containing dialkylaminoalkyl substituents in position 10 exerted significant effects on G361 cells, with IC_50_ values ranging from 72 to 76 µM. However, compound **3** lacked selectivity, as it reduced fibroblast viability to a similar extent. Among the tested derivatives, compound **5**, containing a pyrazine ring with a chlorine substituent, showed the weakest cytotoxic activity. Compound **6**, containing a pyridine ring with an electron-withdrawing nitro substituent at position 10 of the 1,6-diazaphenothiazine system, proved to be the most promising candidate, demonstrating the lowest IC_50_ value against C32 cells (54 µM). At the same time, this compound showed low toxicity towards normal fibroblasts (selectivity index = 3.48). Consequently, its mechanism of action was investigated in greater detail. Mechanistic studies demonstrated that compound **6** markedly disrupted C32 melanoma cell homeostasis, leading to inhibition of proliferation and reduced cell survival. Specifically, treatment with compound **6** induced mitochondrial membrane depolarization, depletion of intracellular reduced thiols, and DNA fragmentation characteristic of apoptosis. Additionally, the formation of DNA double-strand breaks was detected in C32 cells following exposure to compound **6**. These in vitro findings provide a foundation for further investigation into the molecular mechanisms underlying the anticancer activity of phenothiazine derivatives and may support the development of new compounds with improved selectivity toward cancer cells.

## Figures and Tables

**Table 1:** The concentration that reduces viability by 50% (IC_50_, µM) of derivatives **2**–**6** was determined in melanoma cell lines and human dermal fibroblasts (HDF).

	A375	C32	G361	SK-MEL-28	HDF
**2**	104	131	73	106	134
**3**	102	119	76	95	78
**4**	Neg *	168	72	106	Neg *
**5**	Neg *	89	101	Neg *	Neg *
**6**	127	54	98	Neg *	188

* “Neg” indicates that the effect was too low to allow estimation of the IC_50_.

## Data Availability

The original contributions presented in this study are included in the article/Supplementary Materials. Further inquiries can be directed to the corresponding author.

## Supplementary Materials

The following supporting information can be downloaded at: https://www.mdpi.com/article/10.3390/cimb48050490/s1.
