# Supplementary material for: Cytotoxic and Antimelanoma Activity of Selected 3-Methyl-1,6-diazaphenothiazines in Human Melanoma Cells—In Vitro Studies
Source: Curr Issues Mol Biol. 2026 May 9;48(5):490. doi: 10.3390/cimb48050490 (PMC13206610; doi:10.3390/cimb48050490)
Supplement: Supplementary file 1 [file cimb-48-00490-s001.zip › cimb-4243365-supplementary.pdf]

Supplementary Material

Cytotoxic and Antimelanoma Activity of selected 3-Methyl-1,6-Diazaphenothiazines in Human Melanoma Cells - In vitro studies

Beata Morak-Młodawska[1*], Małgorzata Jeleń[1], Zuzanna Rzepka[2*], Milena Koch[2], and Dorota Wrześniok[2]

1 , Department of Organic Chemistry, Faculty of Pharmaceutical Sciences in Sosnowiec, Medical Univer-sity of Silesia, Jagiellońska 4, 41-200 Sosnowiec, Poland, bmlodawska@sum.edu.pl (BMM), manowak@sum.edu.pl (MJ)

2 Department of Pharmaceutical Chemistry, Faculty of Pharmaceutical Sciences in Sosnowiec, Medical University of Silesia, Jagiellońska 4, 41-200 Sosnowiec, Poland; zrzepka@sum.edu.pl (Z.R.); s83400@365.sum.edu.pl (M.K.); dwrzesniok@sum.edu.pl (D.W.)

* Correspondence: bmlodawska@sum.edu.pl (BMM); zrzepka@sum.edu.pl (Z.R.)

Content

1. 1H NMR, 2D NMR, 13C NMR, HR MS of most active compound (**6**)

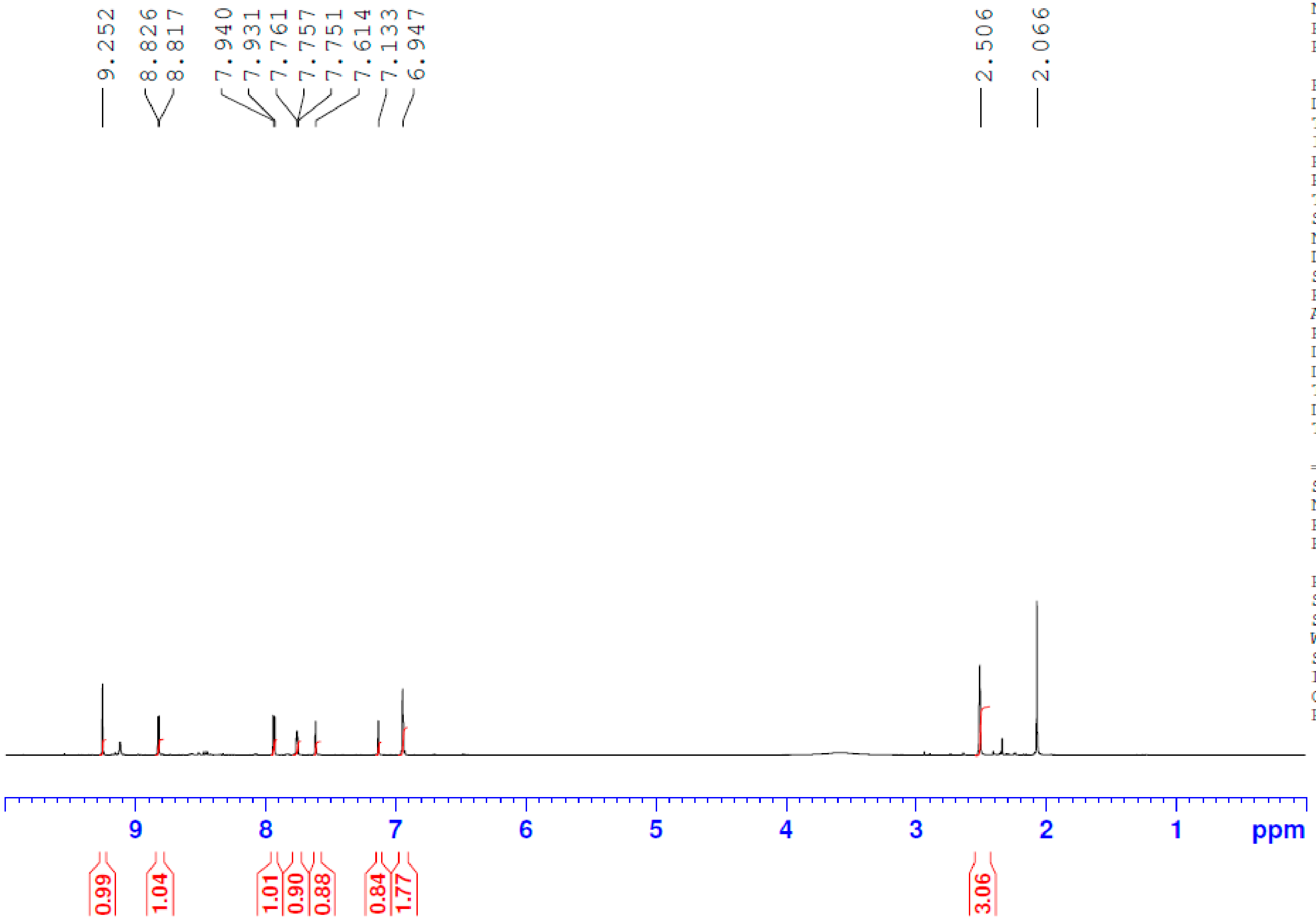


```
Current Data Parameters
NAME          BMJ-D1-2-2-26
EXPNO                     1
PROCNO                    1

F2 - Acquisition Parameters
Date_              20260202
Time                   8.49
INSTRUM               spect
PROBHD      5 mm PABBO BB/
PULPROG                zg30
TD                    65536
SOLVENT                DMSO
NS                       10
DS                        0
SWH               12019.230 Hz
FIDRES             0.183399 Hz
AQ                2.7262976 sec
RG                       32
DW                   41.600 usec
DE                     6.50 usec
TE                    291.9 K
D1               1.00000000 sec
TD0                       1

======== CHANNEL f1 ========
SFO1          600.1637062 MHz
NUC1                   1H
P1                  11.00 usec
PLW1          19.50000000 W

F2 - Processing parameters
SI                    65536
SF            600.1600000 MHz
WDW                      EM
SSB       0
LB                     0.30 Hz
GB        0
PC                     1.00
```

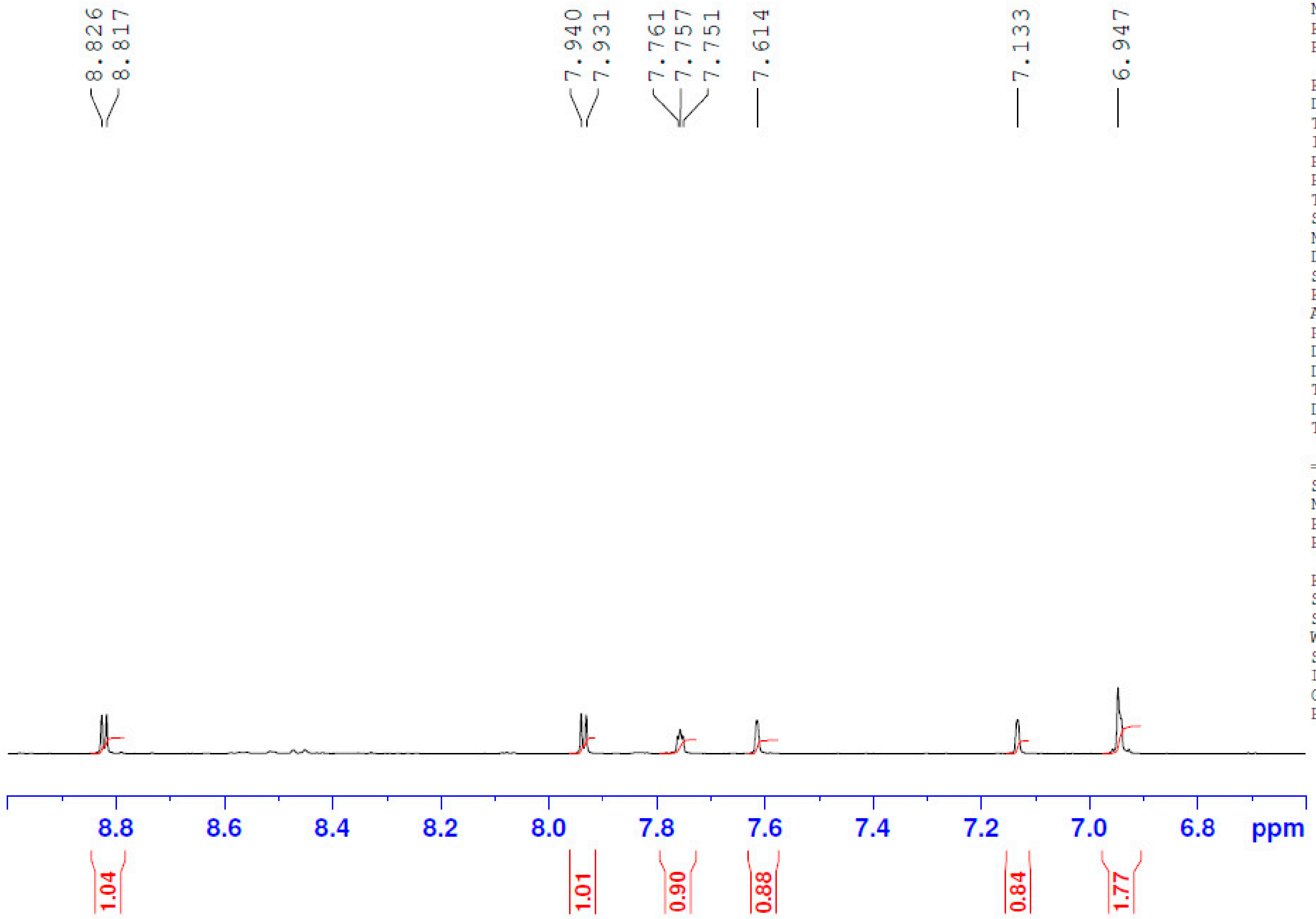

Current Data Parameters
NAME BMJ-D1-2-2-26
EXPNO 1
PROCNO 1

F2 - Acquisition Parameters
Date_ 20260202
Time 8.49
INSTRUM spect
PROBHD 5 mm PABBO BB/
PULPROG zg30
TD 65536
SOLVENT DMSO
NS 10
DS 0
SWH 12019.230 Hz
FIDRES 0.183399 Hz
AQ 2.7262976 sec
RG 32
DW 41.600 usec
DE 6.50 usec
TE 291.9 K
D1 1.00000000 sec
TD0 1

======== CHANNEL f1 ========
SFO1 600.1637062 MHz
NUC1 1H
P1 11.00 usec
PLW1 19.50000000 W

F2 - Processing parameters
SI 65536
SF 600.1600000 MHz
WDW EM
SSB 0
LB 0.30 Hz
GB 0
PC 1.00

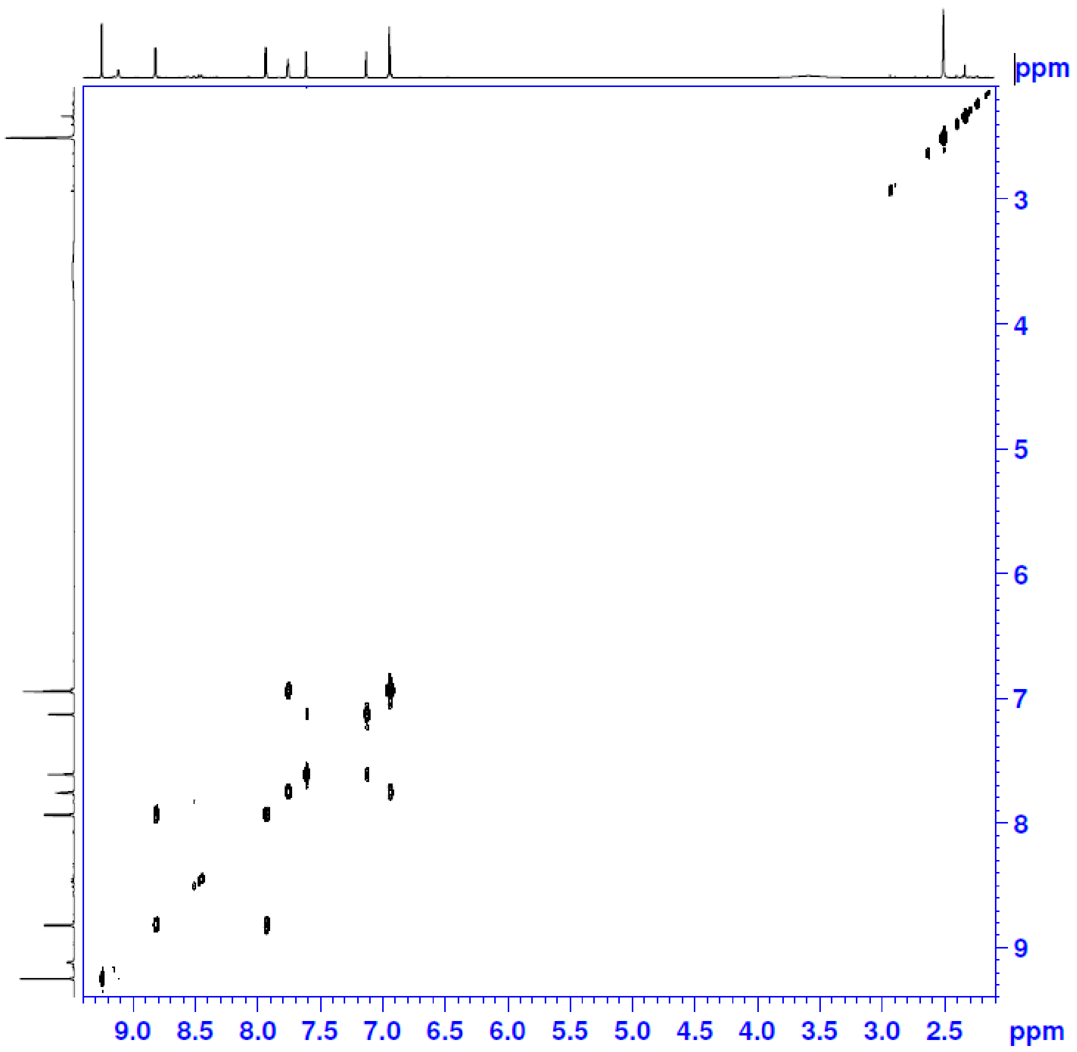
ppm
3
4
5
6
7
8
9
9.0
8.5
8.0
7.5
7.0
6.5
6.0
5.5
5.0
4.5
4.0
3.5
3.0
2.5
ppm

13c

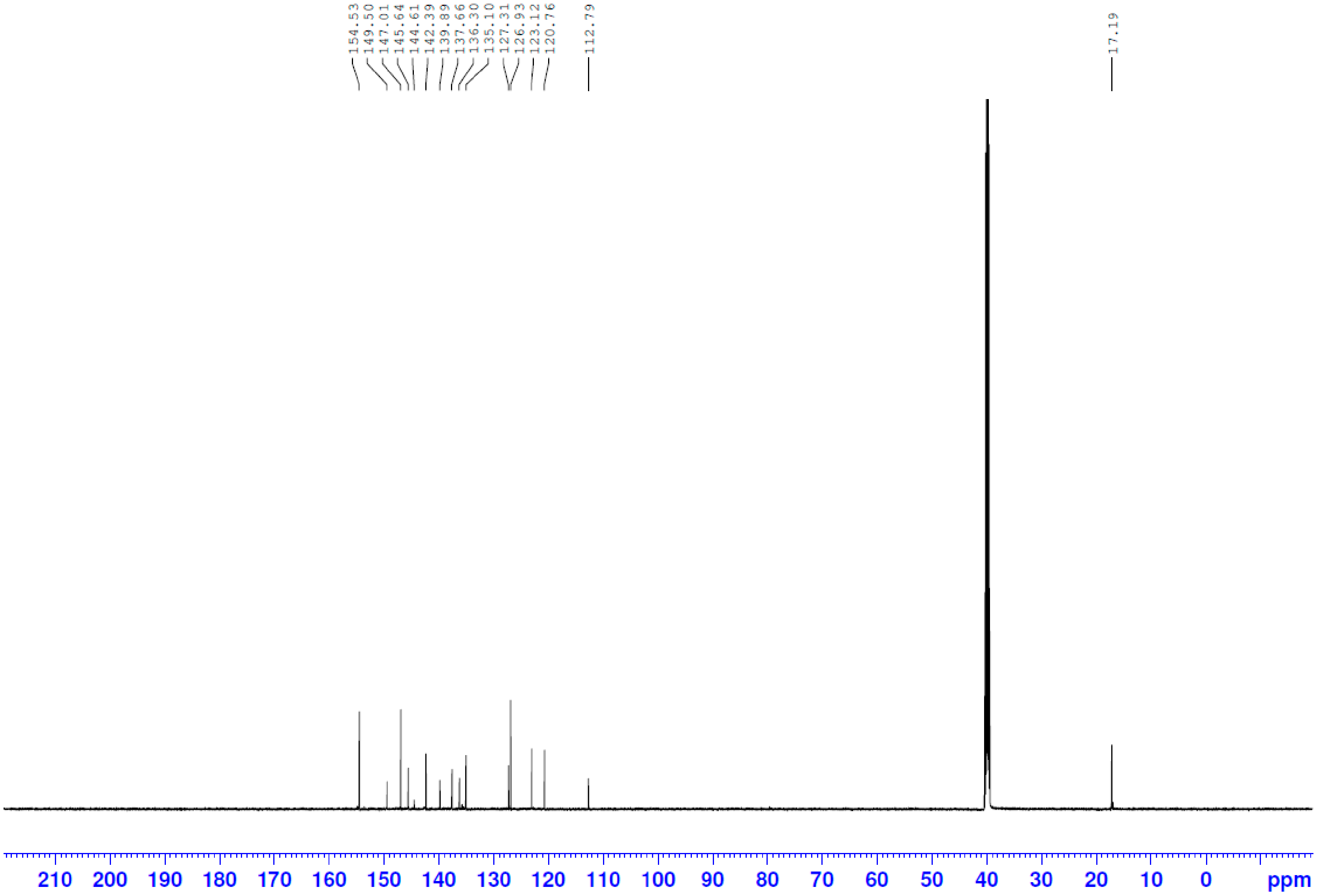
154.53
149.50
147.01
145.64
144.61
142.39
139.89
137.66
136.30
135.10
127.31
126.93
123.12
120.76
112.79
17.19
210 200 190 180 170 160 150 140 130 120 110 100 90 80 70 60 50 40 30 20 10 0 ppm

Analysis Name	D:\Data\BMD2 2024.d			
Method	low_mass_positive.m	Operator	KM	
Sample Name	Tune_neg_high_post	Instrument	impact II	1825265.10082
Comment				

Acquisition Parameter

Source Type	ESI	Ion Polarity	Positive	Set Nebulizer	0.3 Bar
Focus	Active	Set Capillary	4000 V	Set Dry Heater	200 °C
Scan Begin	100 m/z	Set End Plate Offset	-500 V	Set Dry Gas	3.0 l/min
Scan End	1000 m/z	Set Charging Voltage	2000 V	Set Divert Valve	Source
		Set Corona	0 nA	Set APCI Heater	0 °C

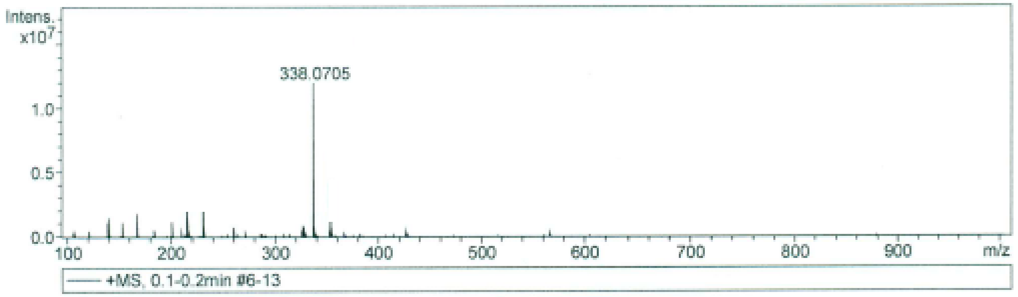

#	m/z	Res.	S/N	I	I %	FWHM
1	338.0705	37628	8056.1	4766423	100.0	0.0090
